# Translation, cross-cultural adaptation, and validation of the Caregiver Indirect and Informal Care Cost Assessment Questionnaire for end-of-life care into Spanish

**DOI:** 10.1017/S1478951525101508

**Published:** 2026-01-13

**Authors:** Laura S. Lamfre, Maria Coller, Clément Meier, Pilar Barenstein-Fonseca, Claudia Fischer, Judit Simon, Vilma Tripodoro

**Affiliations:** 1 Department of Economics, National University of Comahue, Neuquén, Argentina; 2 Cancer Control Program, Advanced Chronicity and Palliative Care, Ministry of Health of Rio Negro, Río Negro, Argentina; 3 Faculty of Business and Economics (HEC), University of Lausanne, and Swiss Centre of Expertise in the Social Sciences (FORS), University of Lausanne (HEC), Lausanne, Switzerland; 4 Instituto Pallium Latinoamérica, Ciudad de Buenos Aires, Argentina; 5 Instituto Cudeca de Estudios e Investigación en Cuidados Paliativos, Fundación Cudeca, Málaga, Spain; 6 Department of Health Economics, Medical University of Vienna, Vienna, Austria; 7 Atlantes, Global Observatory of Palliative Care, University of Navarra, Pamplona, Spain

**Keywords:** Informal care cost, productivity cost, informal care, psychometric validation, adaptation

## Abstract

**Objective:**

This study aims to adapt and validate a Spanish (Argentina) version of the Caregiver Indirect and Informal Care Cost Assessment Questionnaire (CIIQ) to enable the measurement of informal care-related costs in the Argentine context, addressing the current lack of Spanish-language tools for assessing indirect costs.

**Method:**

The CIIQ was translated, cross-culturally adapted, and validated for the Spanish–Argentine language and culture. Psychometric properties were evaluated in a purposive sample of relatives of patients with advanced chronic disease and limited life expectancy in Argentina. Missing data and internal consistency (Cronbach’s *α*) were assessed, along with discriminant capacity, content, and construct validity. Construct validity was examined through principal component analysis (PCA) and confirmatory factor analysis (CFA).

**Results:**

The translation and cultural adaptation process was completed without major difficulties. A total of 154 caregivers completed the baseline questionnaire and 90 completed the follow-up assessment, with missing data remaining below 10% across items. Internal consistency was high for the overall instrument (*α* = 0.802) and for the unpaid care cost domain (*α* = 0.866). The productivity loss domain showed moderate reliability (*α* = 0.362). Low correlations with unrelated domains (*ρ* < 0.2) supported adequate discriminant validity. PCA identified 2 components – informal care costs (51.5% of explained variance) and productivity loss costs (20.3%) – which were further supported by CFA.

**Significance of results:**

The Spanish–Argentine version of the CIIQ is a reliable and culturally appropriate instrument for assessing the economic burden of informal care in Argentina. While the unpaid care items demonstrated strong psychometric performance, productivity-related items may require refinement to improve reliability in future applications.

## Introduction

In recent years, the concept of caregiving has acquired increasing relevance in both theoretical and political discussions (Alliance WPC and Organization WH 2014; OIT, UNICEF, PNUD and CIPPEC 2018; Tronto 2020). Caregiving encompasses the essential tasks required to sustain daily life and ensure the continuity of caregiving roles across generations. It encompasses not only material and physical care but also the concern, affection, and attention provided to individuals in need for a variety of reasons (CEPAL NU 2022). In palliative care, where patients often require extensive and ongoing support, quantifying the informal costs associated with these caregiving practices is essential for understanding the total burden of illness (Gardiner et al. 2014; Landfeldt et al. 2019; Fischer et al. 2024).

Informal caregivers play a crucial role in supporting patients across many disease areas, including chronic conditions and cancer (Fischer et al. 2020). A recent study highlighted the critical role of family caregivers in end-of-life care but also emphasized the need for better support from society, healthcare teams, and family systems (Tripodoro et al. 2024). Despite widespread recognition of their importance, their contributions, in terms of both costs and outcomes, are often overlooked in economic evaluations. Failing to account for these costs can result in a biased analysis of the true costs and benefits of interventions in palliative care. Likewise, the disproportionate burden placed on families, particularly women, in end-of-life care raises concerns regarding both social equity and gender fairness (Rodríguez Enríquez 2015; Cepal NU 2020; CEPAL NU 2022).

Economic evaluations compare costs and outcomes, and adopting a societal perspective is often recommended (Krol et al. 2015). From this perspective, all costs should be considered, regardless of who bears them (Garrison et al. 2018). This means that, in addition to direct medical costs, nonmedical costs – both direct and indirect – must also be included. For example, transportation costs for the patient or informal caregivers, as well as productivity losses due to work absence or reduced efficiency during paid or unpaid work (Drummond et al. 2015). However, many cost-effectiveness studies in palliative care have relied primarily on model-based analyses using secondary data sources, and often lack empirical data directly collected from patients and informal caregivers, particularly regarding nonmedical and caregiving costs (Lamfre et al. 2023a, 2023b, 2024). Although new, internationally harmonized self-reported measures to collect cost information for multi-sectoral costing are now available (Pokhilenko et al. 2023), additional reporting on caregiver costs directly by the caregivers is crucial.

Studies that have incorporated the costs of informal caregiving into economic evaluations in health in Latin America are relatively scarce but demonstrate a growing interest in addressing this critical component of nonmedical costs. In Latin America, Espinola et al. (2023) analyzed the costs associated with the time of informal caregivers for patients with chronic diseases in the region, highlighting their significant economic impact through the opportunity cost approach. Similarly, Díaz et al. (2006) made visible the value of the informal caregivers, emphasizing the essential role of households in health production. The Pan American Health Organization (2008) also advocates for the inclusion of informal care in economic evaluations and provides a methodology to address this challenge, recognizing its substantial contribution to health systems. In Argentina, Lamfre et al. (2023a, 2023b, 2024) examined the economic impact of informal care for cancer patients at the end of life, using robust methodologies to estimate both direct and indirect costs. In Spain, Oliva-Moreno et al. (2015) estimated the economic value of informal care provided to dependent individuals, employing advanced techniques to highlight its significance. Additionally, the National Directorate of Economy, Equality, and Gender of Argentina (2020) quantified the economic contribution of unpaid domestic and care work, underscoring its strategic importance to national economies. However, these studies generally estimated the number of hours dedicated to caregiving theoretically, without applying a structured questionnaire to the target population, which could limit the precision of the estimates. These findings collectively emphasize the critical need to integrate informal care costs into health economic evaluations and to develop public policies that support and recognize the indispensable role of informal caregivers in healthcare systems.

To accurately capture comprehensive caregiver costs in economic evaluations, the Caregiver Indirect and Informal Care Cost Assessment Questionnaire (CIIQ) was developed in 2018 (Landfeldt et al. 2019). This tool assesses caregiver productivity losses and informal care expenses across a range of conditions and settings, and is currently available only in English, German, French, Italian, Danish, Norwegian, Finnish, and Swedish, although no validations and cultural adaptations of this instrument have been found. However, despite its importance, a Spanish-language version of the CIIQ is essential to address the specific cost dynamics of informal caregiving in Spanish-speaking populations, particularly in Argentina.

Producing valid and valuable evidence for the development of palliative care requires that research methods in this field adapt their methodology to the specific conditions of these settings (Fischer et al. 2024). However, there is a lack of a standardized research framework for the methods used in economic evaluations of palliative care interventions, particularly when it comes to assessing the costs of informal care for patients at the end of life.

The main objective of this study was to translate, adapt and validate the CIIQ questionnaire in Argentina, adapting it as a tool specifically designed to assess the indirect and informal care costs of caregivers. This validation will enhance the reliability of health economic evaluations of palliative and end-of-life care interventions in Spanish-speaking countries, ultimately supporting the development of more effective strategies in palliative care.

## Methods

This study is part of the ongoing international project iLIVE, Live Well, Die Well (EU Horizon 2020 ID: 825731), which aims to describe the experiences of patients with advanced, potentially life-threatening chronic illnesses at the end of life and their families in Argentina, Germany, Iceland, the Netherlands, Norway, Slovenia, Spain, Sweden, Switzerland, and the United Kingdom. Details regarding the project’s protocol and implementation can be found elsewhere (Yildiz et al. 2022). One of the specific objectives of this project was to conduct economic evaluations in end-of-life palliative care.

The CIIQ questionnaire, integrated into the international iLIVE project, was translated, cross-culturally adapted, and validated for the Spanish–Argentine language and culture. Psychometric characteristics were explored in a purposive sample of relatives of patients with advanced chronic disease and a short life expectancy.

The CIIQ questionnaire is designed to estimate caregivers’ indirect and informal care costs as separate, mutually exclusive components of total social costs, using valuation methods such as the opportunity cost and proxy good approaches (Landfeldt et al. 2019). For this purpose, questions are designed to collect data on relevant aspects of the past and present employment situation of caregivers of patients receiving palliative care at the end of life to measure the total loss of working time. The questions are designed to capture the number of hours of free time (nonworking hours) dedicated to informal care, in order to estimate the associated costs.

Indirect costs and costs of paid informal care are valued using the human capital approach, consistent with the original CIIQ framework. This method values time losses based on foregone earnings or potential output, providing a straightforward and widely accepted estimate of economic impact. Costs of unpaid informal care can be valued and estimated as the loss of free time quantified using the opportunity cost method or the proxy suitable method (van den Berg et al. 2004; Landfeldt et al. 2019).

The CIIQ questionnaire consists of 13 items and incorporates a follow-up period of 4 weeks. The questions are geared toward recording data on all relevant aspects of past and current work status (including work status, work hours, absenteeism, and productivity while working) to measure total lost work hours. To estimate the cost of informal care, the questions are designed to record the number of hours of free time (i.e., nonwork hours) spent on informal care using the recall method. Four different categories of informal care activities and tasks were included to allow for a more accurate assessment of informal care based on the replacement cost method: (1) household activities, (2) personal care, (3) practical support, and (4) emotional support. The questionnaire was also formulated to record data related to paid informal care, i.e., when the caregiver receives financial compensation to care for the patient.

### Design and translation

The process of translation and cultural adaptation of the CIIQ form was carried out following international guidelines to guarantee conceptual and linguistic equivalence (Beaton et al. 2000; Wild et al. 2005). The translation process into Spanish was developed for Argentina, including forward and backward translation, expert committee review, and cognitive pre-testing, ensuring semantic equivalence and cultural adequacy. The adapted form, presented in Supplementary material, was administered in a pilot study with a representative sample of 9 Argentinian relatives to evaluate its validity and reliability. The results of this pilot study provided the basis for determining the effectiveness of the cultural fit and the internal consistency of the form in its new version.

#### Participants

The psychometric characteristics of the CIIQ were explored in a sample composed of competent adult patients with an estimated life expectancy of 6 months or less, regardless of sex, age, place of residence, or type of care. These patients were eligible, and their family members/informal caregivers were invited to participate with the patient’s consent. In 7 Argentinian healthcare centers including the Pallium Latin America Institute, the Lanari Medical Research Institute (University of Buenos Aires), the CB Udaondo Gastroenterology Hospital, the C. Argerich Acute Care Hospital, Del Parque Center, the Baires CCP, all based in the City of Buenos Aires, and the Private University Hospital of Cordoba in Cordoba City, the adapted Surprise Question (White et al. 2017) was used to assess whether the treating physician would be surprised if a patient died within 6 months. Additionally, the SPICT tool criteria (Highet et al. 2014) were applied to identify eligible patients with a short life expectancy (Yildiz et al. 2022). These patients were invited to participate, provided informed consent, and were interviewed in person to explore their preferences, values, and perspectives on end-of-life decisions. They were also asked for permission to invite a relative to participate.

At baseline, informal caregivers included in the study completed the CIIQ as part of a comprehensive questionnaire that also covered sociodemographic and economic variables, caregiving time, caregiving burden, quality of life, and health-related quality of life. After 30 days, the same questionnaire was re-administered to the same caregivers, following prior informed consent. A visual analogue scale from 0 (not at all straining) to 10 (much too straining) was used to assess the caregiving burden. Quality of life was also evaluated using the EORTC QLQ C15 PAL visual analogue scale (Groenvold et al. 2006) with values from 1 (very poor) to 7 (excellent). The EuroQol-5D-5L (Stolk et al. 2019) form was used to measure health-related quality of life. This standardized assessment tool is designed to capture the impact of different health states on 5 key dimensions: mobility, self-care, usual activities, pain/discomfort, and anxiety/depression. Each dimension is classified into 5 levels of severity, from “no problems” to “extreme problems.”

The study was approved by the institutions’ ethics committees where it was conducted. The protocol numbers were omitted for double-blind review. It started in March 2020 and ended in May 2023.

### Analysis

All analyses were conducted using SPSS software version 24, except the confirmatory factor analysis (CFA), which was performed using R-project 4.3.3 software. The database consisted of responses to the CIIQ form from informal caregivers. The proportion of missing data in the database at baseline and follow-up was analyzed to assess the feasibility and quality of information.

A descriptive analysis of the questionnaire responses at baseline and at 4-week follow-up was conducted. Indicators of caregiver indirect costs (loss of productivity in paid work) and caregiver informal care costs (unpaid informal care) were calculated following the methodology outlined by Landfeldt et al. (2019) in Tables 1 and 2, using the CIIQ.

**Table 1.** Demographic characteristics and clinical status of caregivers

	Baseline (*n* = 154)		Follow-up (*n* = 90)	
	Values	Missing *n* (%)	Values	Missing *n* (%)
Female, *n* (%)	109 (70.8)	1 (0.6)	66 (74.2)	1 (1.1)
Age in years, mean (SD)	53.7 (14.5)	10 (6.5)	54.3 (14.3)	5 (5.5)
University educational level, *n* (%)	60 (39.2)	1 (0.6)	36 (40.0)	0 (0)
Argentine nationality, *n* (%)	140 (90.9)	5 (3.2)	81 (90.0)	0 (0)
Living with a partner, *n* (%)	48 (31.2)	0 (0)	29 (32.2)	0 (0)
Belonging to a particular religious, spiritual, philosophical group, *n* (%)	68 (44.2)	2 (1.3)	45 (50.6)	1 (1.1)
Income level (USD), *n* (%)	–	4 (2.6)	–	0 (0)
<1000	27 (18.00)	–	19 (21.1)	–
1000–1999	40 (26.67)	–	23 (25.6)	–
2000–2999	21 (14.00)	–	8 (8.89)	–
3000–3999	9 (6.00)	–	5 (5.56)	–
>4000	17 (11.33)	–	11 (12.22)	–
I prefer not to answer this question	36 (24.0)	–	24 (26.7)	–
Your familiar receives professional care or support at home, *n* (%)	45 (29.6)	2 (1.3)	30 (34.5)	3 (3.3)
You have been caring for your family member for more than a year, *n* (%)	82 (53.9)	2 (1.3)	59 (66.3)	1 (1.1)
Burden currently on caring for your family member, *n* (%)	–	1 (0.6)	–	3 (3.3)
0 (no at all straining)	41 (26.6)	–	16 (18.4)	–
1	6 (3.9)	–	5 (5.7)	–
2	4 (2.6)	–	5 (5.7)	–
3	9 (5.8)	–	3 (3.4)	–
4	8 (5.2)	–	5 (5.7)	–
5	17 (11.0)	–	6 (6.9)	–
6	6 (3.9)	–	10 (11.5)	–
7	19 (12.3)	–	9 (10.3)	–
8	18 (11.7)	–	13 (14.9)	–
9	8 (5.2)	–	5 (5.7)	–
10 (much too straining)	17 (11.0)	–	10 (11.5)	–
Rating your quality of life over the past week	–	3 (1.9)	–	3 (3.3)
1 (very poor)	5 (3.3)	–	5 (5.7)	–
2	7 (4.6)	–	2 (2.3)	–
3	20 (13.2)	–	9 (10.3)	–
4	27 (17.9)	–	20 (23.0)	–
5	48 (31.8)	–	26 (29.9)	–
6	25 (16.6)	–	16 (18.4)	–
7 (excellent)	19 (12.6)	–	9 (10.3)	–
Health-related quality of life today, mean (SD)	72.4 (16.4)	2 (1.3)	68.3 (18.6)	1 (1.1)
EuroQoL-5D-5L1: no mobility problems, *n* (%)	133 (86.9)	1 (0.6)	74 (82.2)	0 (0)
EuroQoL-5D-5L1: no washing issues and dressing on their own, *n* (%)	146 (95.4)	1 (0.6)	82 (91.1)	0 (0)
EuroQoL-5D-5L1: no problems performing daily activities, *n* (%)	121 (79.1)	1 (0.6)	64 (71.1)	0 (0)
EuroQoL-5D-5L1: no pain or discomfort, *n* (%)	66 (43.1)	1 (0.6)	37 (41.6)	1 (1.1)
EuroQoL-5D-5L1: no anxiety or depression, *n* (%)	34 (22.2)	1 (0.6)	26 (29.2)	1 (1.1)

*n* = Number of respondents; SD = standard seviation; USD = United States Dollars; EuroQol-5D-5L1 = The 5-level version of the EuroQol 5-Dimension health questionnaire.

**Table 2.** Descriptive statistics of the main items of the CIIQ questionnaire and indicator scores

	Baseline assessment				Four-week follow-up assessment			
	*N*	Range	Mean	SD	*N*	Range	Mean	SD
2. How many hours a week do you work?	90	(3–105)	37.0	16.6	52	(4–72)	33.4	18.3
5. How many hours a week did you work before reducing your working hours?	20	(0–105)	37.0	25.0	10	(4–72)	39.7	17.4
6. During the last week, how many hours have you missed work due to your family member’s illness/condition?	75	(0–60)	8.9	12.5	43	(0–40)	7.2	11.0
7. During the last week, how much did your family member’s illness/condition affect your productivity at work?	93	(0–10)	4.1	3.2	57	(0–10)	3.5	3.1
8. If you stopped working because of your family member’s illness/condition, how many hours did you work per week?	11	(6–70)	26.8	19.0	7	(6–48)	28.4	16.5
10. During the past week, how much time have you spent on household and other tasks that you would not have to do if your family member were in good health, or if you could have done them independently?	130	(0–168)	22.3	36.8	75	(0–100)	16.3	22.3
11. During the last week, how much time have you spent helping your family members with their care?	123	(0–168)	19.3	40.9	67	(0–168)	12.2	29.5
12. During the last week, how many hours have you spent giving practical support to your family member that you would not have to do if your family member was in good health or if you could have done it independently?	132	(0–168)	17.8	37.4	77	(0–168)	20.0	37.3
13. During the last week, how much time have you spent giving emotional support to your family member that you would not have to do if your family member was in good health?	146	(0–168)	35.5	52.6	87	(0–168)	24.6	41.5
***Indicator scores***^a^								
Loss of productivity paid work (hours/year)	83	(132 –5145)	1210	847	50	(59–4096)	1068	786
Unpaid informal care (hours/year)	148	(52–6552)	2972	2288	85	(52–6552)	2506	2086

a Calculated following Landfeldt et al. (2019) methodology.

The instrument’s internal consistency, which refers to the homogeneity of items measuring the same attribute, was assessed using Cronbach’s *α* coefficient. This coefficient evaluates the correlation between the items in the questionnaire, determining how consistently different items function as part of the same measurement construct (Terwee et al. 2007). In the present study, the CIIQ captures 2 components of social costs: loss of productivity in paid work and the cost of unpaid informal care. Accordingly, Cronbach’s *α* coefficient was calculated separately for the overall social cost and each component. While each item is necessary to estimate costs according to the original CIIQ methodology – where items constitute non-redundant data points – the psychometric assessment of internal consistency provides complementary information on the reliability of the instrument as a measurement tool. This approach does not imply any modification or exclusion of items for cost estimation purposes but aims to support future refinement of the instrument and to better understand how consistently the items reflect the underlying construct in different settings.

Another important characteristic to analyze in instruments evaluating multidimensional scales is their discriminant power, which refers to the ability to determine whether the items within each domain measure only the concepts of that specific dimension, without overlapping with those of other dimensions (Campo-Arias and Oviedo 2008; Luján-Tangarife and Cardona-Arias 2015). Discriminant power was assessed by calculating Pearson correlation coefficients between items and the domains to which they do not belong, thereby determining the success percentage for each domain.

Construct validity, which assesses whether the instrument’s items adequately represent the construct it aims to measure (Luján-Tangarife and Cardona-Arias 2015), was evaluated through exploratory factor analysis (EFA), specifically with principal component analysis (PCA). This analysis aimed to determine whether the underlying dimensions (components) in the instrument align with the construct to be measured. To test the factor model fit and item adequacy, Bartlett’s test of sphericity (Bartlett 1954) and the Kaiser–Meyer–Olkin (KMO) test (Kaiser 1974) were applied. Values greater than 0.7 for these tests are considered satisfactory (Pérez Gil et al. 2000; Batista-Foguet et al. 2004).

To assess the structural factors, CFA was performed. Specifically, a model was developed to assess the goodness of fit of the social cost structure, which consists of 2 factors: productivity loss and unpaid informal care costs. The goodness of fit to the model was examined by the chi-square and Comparative Fit Index (CFI).

## Results

### Translation and cross-cultural adaptation of the CIIQ

Several issues were carefully analyzed during the translation and cross-cultural adaptation process, including the cognitive debriefing stage. Adjustments were made to the phrasing and content of the CIIQ to ensure cultural relevance and clarity for the Argentine context. These modifications aimed to align the questionnaire with local cultural norms and linguistic nuances while maintaining its conceptual integrity.

During the first step of translating the questionnaire from English to Spanish, certain phrases presented challenges. For instance, “work status” was translated as “employment situation” to better align with the intended meaning. Similarly, the expression “working for pay” posed potential issues, as a direct semantic translation from English could result in inaccuracies. The creation of a reconciled version from the 2 forward translations, corresponding to steps 2 and 3, did not present significant difficulties. Some differences between the translations were identified but were easily reconciled. In relation to the comparison of the backward translations with the original English version, some differences were identified regarding the use of specific terms, which did not pose significant challenges. In particular, these included variations such as “questions/issues,” “with regard to/in relation to,” “skip to/go to,” “disease/illness,” “perform/accomplished,” “as carefully as usual/as well as usual,” “toilet/bathroom,” “providing/giving,” and “taking care/taking charge,” which are considered synonyms in Spanish. The cultural adaptation did not result in significant modifications to the questionnaire.

### Survey sample characteristics

At baseline, 154 caregivers agreed to answer the questionnaire, while the number dropped to 90 at 30-day follow-up. Table 1 presents the demographic characteristics of caregivers.

The majority of caregivers were women (70.8%) of Argentine nationality (90.9%) and in paid employment (63.2%). About 39% had a university education, and 45% reported a monthly income below USD 2000.

### Descriptive analysis

The proportion of missing data was relatively low, less than 10% for all items (ranging from 0% to 6.5%). The continuous variables and calculated indicators of the CIIQ showed a skewed distribution, as they were not symmetrically distributed. Table 2 presents the main descriptive statistics of the CIIQ responses.

### Internal consistency

The questionnaire demonstrated good internal consistency overall, with a Cronbach’s *α* value of 0.802. When analyzed for each component separately, the coefficient was low for the loss of productivity (0.362) and high for unpaid care costs (0.866).

### Discriminating power

Table 3 presents the results for construct discriminant power and divergent validity. In all cases, a low correlation (less than 0.2) was observed between the CIIQ domains and the health-related variables measured by the questionnaire. This finding supports the hypothesis that each domain is calculated independently of variables from other dimensions, confirming the questionnaire’s adequate discriminant power.

**Table 3.** Correlation between CIIQ domains and other health-related variables

	*N*	Correlation coefficient	(*p*-Value)
**Lost productivity**			
How would you rate your overall quality of life over the past week?	81	−0.01	0.97
EuroQoL-5D-5L1: your health status today: Mobility	83	0.08	0.47
EuroQoL-5D-5L1: your health status today: self-care	83	−0.09	0.44
EuroQoL-5D-5L1: your health status today: daily activities	83	0.01	0.93
EuroQoL-5D-5L1: your health status today: pain/discomfort	83	0.02	0.84
EuroQoL-5D-5L1: your health status today: anxiety/depression	83	−0.06	0.61
What is your health status today on a scale of 0–100?	83	−0.02	0.88
**Cost of informal care**			
How would you rate your overall quality of life over the past week?	146	0.08	0.34
EuroQoL-5D-5L1: your health status today: mobility	148	−0.00	0.96
EuroQoL-5D-5L1: your health status today: self-care	148	−0.01	0.88
EuroQoL-5D-5L1: your health status today: daily activities	148	0.02	0.83
EuroQoL-5D-5L1: your health status today: pain/discomfort	148	0.12	0.17
EuroQoL-5D-5L1: your health status today: anxiety/depression	148	−0.05	0.54
What is your health status today on a scale of 0–100?	147	0.02	0.80

### Construct validity

The EFA demonstrates that the items included are relevant and cover all domains of lost productivity and unpaid care costs. This analysis shows a solution with 2 main components that explain 71.8% of the total variance of the data. The first component explains 51.5% and the second 20.3% of the total variance. In particular, the first component defines the costs of informal care since questions 10, 11, 12 and 13 are the ones that have significant loadings in this component. In contrast, the second component represents the costs of lost productivity at work, with relevant loads of questions 6 and 7 (Table 4). Bartlett’s test of sphericity for the PCA yielded a significance level of 0.00, indicating that the dataset is suitable for data reduction analysis. Likewise, the KMO indicator was 0.751, which is considered satisfactory for conducting this analysis.

**Table 4.** Factor loading of questions on components extracted from the PCA

Matrix of rotated components		
	Component	
	1	2
2. How many hours a week do you work?		
6. During the last week, how many hours have you missed work due to your family member’s illness/condition?		0.82
7. During the last week, how much did your family member’s illness/condition affect your productivity at work?		0.89
10. During the past week, how much time have you spent on household and other tasks that you would not have to do if your family member were in good health, or if you could have done them independently?	0.93	
11. During the last week, how much time have you spent helping your family members with their personal care?	0.93	
12. During the last week, how many hours have you spent giving practical support to your family member that you would not have to do if your family member was in good health or if you could have done it independently?	0.94	
13. During the last week, how much time have you spent giving emotional support to your family member that you would not have to do if your family member was in good health?	0.83	

Extraction method: PCA. Rotation method: Varimax normalization with Kaiser. Only loads greater than 0.4 are reported.

**Figure 1.**
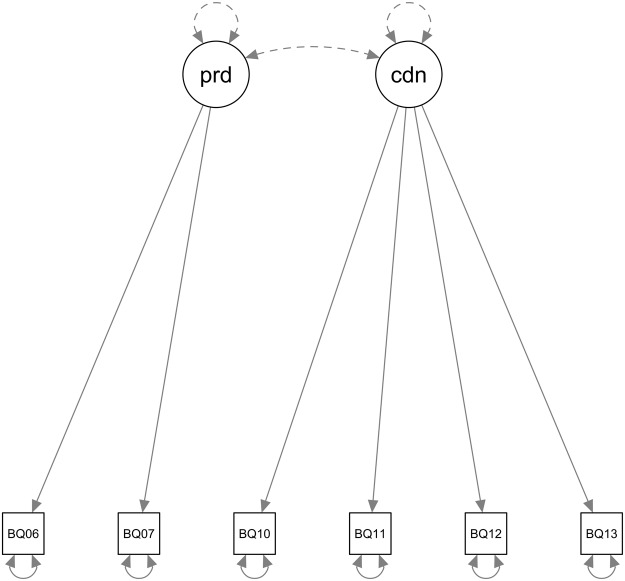
Confirmatory Factor Analysis (CFA) model with five variables and two factors.

CFA was conducted to examine the structural validity of the proposed domains. The result confirms that items 6 and 7 allow for the estimation of the indirect costs of productivity loss, while items 10–13 estimate the costs of informal care. The flow chart of the CFA model is shown in Figure 1. The goodness of fit was evaluated using the *χ*^2^ statistic, which presented a significance value of 0.024. Likewise, the CFI was 0.966, indicating that the model adequately fits the data.

## Discussion

In this study, we evaluated the measurement properties of the Argentine Spanish version of the CIIQ. The overall results are considered reasonable based on data quality analysis, content and construct validity, and reliability.

The CIIQ questionnaire was designed to collect data for assessing and estimating the cost of care components, regardless of the patient’s disease, condition, or geographic location. It is a relatively new tool with only 1 prior study conducted using it (Landfeldt et al. 2019). The CIIQ has yet to be validated and cross-culturally adapted for the Spanish language. It is currently available in English, German, French, Italian, Danish, Norwegian, Finnish, and Swedish (“The CIIQ” n.d.).

The translation and cross-cultural adaptation of the CIIQ for Argentina were completed with minimal challenges. Minor issues arose with specific terms, which were resolved to ensure accurate meaning. The reconciliation of forward translations and comparison with the original text revealed minor discrepancies, which did not require significant modifications. Overall, the process confirmed the questionnaire’s cultural suitability for the Argentine context.

The findings of this study align with previous research on the cross-cultural validation and adaptation of questionnaires related to productivity costs and informal care, such as the Al-Aqeel et al. (2021) paper that adapts the iMTA Productivity Cost Questionnaire (iPCQ) to the Arab context.

The reliability of the adapted questionnaire is supported by the low percentage of missing data, indicating that respondents were able to understand and complete the items effectively, suggesting good acceptability. On average, the questionnaire took approximately 10–15 minutes to complete, indicating that it is feasible for use in routine care or research settings. The low rate of missing data aligns with findings from validation studies of comparable instruments, such as the study by Kim et al. (2020), which reported a high response rate for the Korean version of the iPCQ.

The global Cronbach’s *α* coefficient demonstrates good internal consistency overall. However, the low internal consistency (Cronbach’s *α* = 0.362) observed in the subdomain of lost productivity costs may be due to the limited number of items, high variability in caregiver employment situations, and the complexity of measuring productivity loss in end-of-life care. These factors suggest that refining existing questions or adding context-sensitive items could improve reliability. Future studies should explore these adjustments to strengthen this component. Similar challenges were noted by Munk et al. (2019) in their validation of the iPCQ for patients with musculoskeletal disorders in Norway, where productivity-related costs yielded less robust results.

The low correlation between the CIIQ domains and the other health variables measured by the questionnaire shows the indicators’ adequate discriminating power, supporting the hypothesis that each domain is calculated independently of different dimensions. These findings align with the results of Kim et al. (2020), who observed a similar correlation between the dimensions assessed when comparing the variables of the IPCQ questionnaire with those of the Short Form-36 Health Survey (SF-36).

PCA revealed a 2-factor structure, consistent with previous research showing that informal care costs and productivity costs tend to group into separate components. For example, Munk et al. (2019) identified a similar factor structure in their adaptation of the iPCQ in Norway, where care-related costs and productivity emerged as distinct factors. Furthermore, this study reinforces the structural validity of the adapted questionnaire. The adequacy indicators, the Bartlett’s sphericity test and the significance of the CFA results provide additional evidence supporting its structural validity.

An additional finding is that most caregivers (70.8%) were women, highlighting the need to analyze the impact of informal caregiving through a gender lens. Various studies have shown that women disproportionately take on caregiving responsibilities, affecting their physical, mental, and economic well-being (Organización Panamericana de la Salud 2008; Folbre 2012). This unequal distribution also reflects indirect and productivity costs, as unpaid caregiving largely falls on women, exacerbating gender disparities in the labor market and social spheres (Espinola et al. 2023).

One of the main limitations of this study is the absence of a comparison between the CIIQ and other instruments or data sources that measure similar constructs. Although a universally accepted gold-standard instrument for assessing indirect costs and informal care is lacking, future studies could consider validating CIIQ responses against national administrative datasets-such as medical absenteeism records-to further support its accuracy. Additionally, while the current sample provides an initial estimate of the questionnaire’s validity and reliability, it was drawn exclusively from Argentina’s 2 largest provinces. Expanding the sample to include participants from more diverse regions could enhance the precision and improve the generalizability of the findings.

One factor that significantly influences informal care costs is the number of hours of paid care a patient receives. Financial constraints often lead to lower-income patients depending more heavily on informal care, while higher-income individuals are more likely to access paid or formal services, thereby reducing the caregiving burden on families and mitigating negative impacts on caregivers’ health and well-being (Brandt et al. 2022). Although the CIIQ includes an item to capture paid informal care (Question 9), in our sample, only three participants reported receiving financial compensation, which limited our ability to analyze this aspect. Future research with larger and more socioeconomically diverse samples could further explore this dimension to provide a more comprehensive understanding of equity-related differences in caregiving costs.

## Conclusions

The results of this study indicate that the adapted version of the CIIQ questionnaire for Argentina is of adequate quality to effectively measure informal care costs and loss of productivity. Therefore, this questionnaire can be used to assess the indirect costs of caregivers and informal caregiving, contributing to the development of more effective strategies in palliative care in Spanish-speaking countries. However, future research could focus on enhancing the internal consistency of the productivity items within this domain. Such improvements would further strengthen the questionnaire’s utility in capturing the specific economic aspects of informal care and the losses experienced by caregivers of patients at the end of life.

## Supporting information

10.1017/S1478951525101508.sm001 Lamfre et al. supplementary material Lamfre et al. supplementary material

## Supplementary material

The supplementary material for this article can be found at https://doi.org/10.1017/S1478951525101508.
