# Supplementary material for: Translation, cross-cultural adaptation, and validation of the Caregiver Indirect and Informal Care Cost Assessment Questionnaire for end-of-life care into Spanish
Source: Palliat Support Care. 2026 Jan 13;24:e24. doi: 10.1017/S1478951525101508 (PMC13166414; doi:10.1017/S1478951525101508)
Supplement: Lamfre et al. supplementary material [file S1478951525101508sup001.pdf]

The Caregiver Indirect and Informal Care Cost Assessment Questionnaire (CIIQ)

Spanish (Argentina) Version 1.0

REVIEW COPY

Copyright © Erik Landfeldt 2025

Full license terms available at http://www.theciiq.com

Cuestionario Evaluación de Costo Indirecto y del Cuidado informal del cuidador (QIIC)

Standardized Questionnaire for the Measurement, Valuation, and Estimation of Costs of Informal Care Based on the Opportunity Cost and Proxy Good Method. Landfeldt et al. Applied Health Economics and Health Policy (2019) 17:15–24

Vamos a preguntarle ahora por temas relacionados con su situación laboral actual y el tiempo que emplea en el cuidado de los niños, pareja, familiares o amigos. Por favor, tenga en cuenta que a partir de ahora nos referiremos a la persona a su cuidado como "familiar", aunque su relación sea diferente.

Situación laboral

1.- ¿En este momento está empleado o es autónomo (trabajo remunerado)?

Si……….. No……… (vaya a la pregunta 8)

2.- ¿Cuántas horas a la semana trabaja? (por ejemplo en relación a su contrato de trabajo) ...…………………….horas por semana

3.- ¿Está trabajando actualmente a tiempo completo?

Si……….. (vaya a la pregunta 6) No……….

4.- ¿Ha reducido su jornada laboral debido a la enfermedad/condición de su familiar? (por ej, para cuidar de él o ella)

Si………. No………… (vaya a la pregunta 6)

5.- ¿Cuántas horas a la semana trabajaba antes de reducir su jornada laboral?

…………………..horas por semana

6.- ¿Durante la última semana, cuántas horas ha perdido de trabajo debido a la enfermedad/condición de su familiar? Se incluyen las horas que ha perdido si ha llegado tarde o tuvo que retirarse antes para acompañar a su familiar a un turno con el médico, al hospital, o a una clínica, ayudarle a vestirse, arreglarse, comer o tomar la medicación.

…………………..horas por semana

REVIEW COPY

7.- ¿Durante la última semana, cuánto ha afectado la enfermedad/condición de su familiar a su productividad mientras estaba trabajando? Si usted pudo trabajar como de costumbre, elija un número bajo. Si no ha sido capaz de trabajar como de costumbre (ej. Ha rendido menos de lo habitual, no se pudo concentrar o realizar determinadas tareas como de costumbre) elija un número alto.

0	1	2	3	4	5	6	7	8	9	10
**Pude trabajar como siempre**<br>**No pude trabajar nada**										

Vaya a la pregunta 9

8.- Si no trabaja: ¿Dejó de trabajar debido a la enfermedad/condición de su familiar?

Sí. ¿Cuántas horas trabajaba a la semana?..............horas por semana

No………….

9.- ¿Está compensado de alguna manera por el tiempo que dedica al cuidado de su familiar? Esto incluye el pago por parte del estado o del seguro para el cuidado de su familiar.

Sí. ¿Cuántas horas a la semana le pagan por el cuidado de su familiar?..............horas por semana

No……….

Cuidado informal

10.- Durante la última semana, ¿cuánto tiempo ha empleado en tareas domésticas y otras que no tendría que realizar si su familiar tuviera buena salud, o si pudiera haberlas hecho de manera independiente? Por ejemplo, preparar la comida, limpiar, lavar, planchar, comprar, jardinería.

………..…………..hs por semana

REVIEW COPY

11.- Durante la <u>última semana</u>, ¿cuánto tiempo ha dedicado a ayudar a su familiar con su cuidado personal? Por ejemplo, vistiéndolo/desvistiéndolo, bañándolo/ duchándolo/ aseándolo, afeitándolo, arreglándole el pelo, llevándolo al al baño.

………..…………..hs por semana

12.- Durante la <u>última semana</u>, ¿cuántas horas ha dedicado a dar apoyo práctico a su familiar que no tendría que realizar si su familiar tuviera buena salud o si pudiera haberlo hecho independientemente? Por ejemplo, darle de comer o beber, sacarlo a la calle (ayudarle a andar o llevarlo en silla de ruedas), llevarlo a visitar a familiares o amigos, acompañarlo a visitas médicas, a la farmacia, ayudarlo con la toma de medicación, hacerse cargo de sus asuntos financieros (pago de facturas, o manejo seguro médico).

………..…………..hs por semana

13.- Durante la <u>última semana</u>, ¿cuánto tiempo ha dedicado a dar apoyo emocional a su familiar que no tendría que realizar si su familiar tuviera buena salud? Por ejemplo, ayudarlo a afrontar el dolor, la discapacidad, el malestar, la ansiedad y las preocupaciones.

………..…………..hs por semana

"The Caregiver Indirect and Informal Care Cost Assessment Questionnaire
– http://www.theciiq.com – Copyright © Erik Landfeldt 2025"

REVIEW COPY
